# Migration pattern, actin cytoskeleton organization and response to PI3K-, mTOR-, and Hsp90-inhibition of glioblastoma cells with different invasive capacities

**DOI:** 10.18632/oncotarget.16847

**Published:** 2017-04-05

**Authors:** Simon Memmel, Dmitri Sisario, Caren Zöller, Vanessa Fiedler, Astrid Katzer, Robin Heiden, Nicholas Becker, Lorenz Eing, Fábio L.R. Ferreira, Heiko Zimmermann, Markus Sauer, Michael Flentje, Vladimir L. Sukhorukov, Cholpon S. Djuzenova

**Affiliations:** ^1^ Department of Radiation Oncology, University Hospital of Würzburg, Würzburg, Germany; ^2^ Department of Biotechnology and Biophysics, University of Würzburg, Würzburg, Germany; ^3^ Fraunhofer-Institut für Biomedizinische Technik, Sulzbach, Germany; ^4^ Molekulare und Zelluläre Biotechnologie/Nanotechnologie, Universität des Saarlandes, Saarbrücken, Germany

**Keywords:** single-cell tracking, dSTORM, super-resolution microscopy, PTEN p53, wound healing

## Abstract

High invasiveness and resistance to chemo- and radiotherapy of glioblastoma multiforme (GBM) make it the most lethal brain tumor. Therefore, new treatment strategies for preventing migration and invasion of GBM cells are needed. Using two different migration assays, Western blotting, conventional and super-resolution (*d*STORM) fluorescence microscopy we examine the effects of the dual PI3K/mTOR-inhibitor PI-103 alone and in combination with the Hsp90 inhibitor NVP-AUY922 and/or irradiation on the migration, expression of marker proteins, focal adhesions and F-actin cytoskeleton in two GBM cell lines (DK-MG and SNB19) markedly differing in their invasive capacity. Both lines were found to be strikingly different in morphology and migration behavior. The less invasive DK-MG cells maintained a polarized morphology and migrated in a directionally persistent manner, whereas the highly invasive SNB19 cells showed a multipolar morphology and migrated randomly. Interestingly, a single dose of 2 Gy accelerated wound closure in both cell lines without affecting their migration measured by single-cell tracking. PI-103 inhibited migration of DK-MG (*p53* wt, *PTEN* wt) but not of SNB19 (*p53* mut, *PTEN* mut) cells probably due to aberrant reactivation of the PI3K pathway in SNB19 cells treated with PI-103. In contrast, NVP-AUY922 exerted strong anti-migratory effects in both cell lines. Inhibition of cell migration was associated with massive morphological changes and reorganization of the actin cytoskeleton. Our results showed a cell line-specific response to PI3K/mTOR inhibition in terms of GBM cell motility. We conclude that anti-migratory agents warrant further preclinical investigation as potential therapeutics for treatment of GBM.

## INTRODUCTION

Glioblastoma multiforme (GBM) is a high-grade astrocytoma associated with a median survival time of 15 months, even after surgical resection, chemotherapy and radiotherapy [1]. Although more long-term survivors have been reported after combined chemo-radiotherapy, the high aggressiveness of GBM caused mainly by the diffuse infiltration of single tumor cells into the surrounding brain parenchyma makes complete tumor debulking virtually impossible [2, 3]. Therefore, better therapies for GBM patients are necessary, involving strategies to prevent cell migration and invasion, which would enhance the tumor response to local treatment. These challenges have led to extensive efforts in elucidating the regulatory mechanisms of GBM cell motility *in vitro* and *in vivo*.

The mechanisms underlying GBM invasion in respect to molecular gene signatures are still unclear [4]. A typical glioblastoma harbors more than 60 genetic alterations [5]. The affected genes/pathways include, most notably, the dysregulated receptor tyrosine kinase (RTK), phosphatase and tensin homologue (*PTEN*), PI3K and inactivated p53 pathways. Frequent mutations in *RTK*, *PTEN* and *p53* genes contribute to a radio- and chemoresistant phenotype and also correlate with poor clinical outcome [6].

The tumor suppressor *PTEN* is deleted or mutated in 30% of GBMs and at lesser frequencies in other tumors [7]. *PTEN* inhibits cell migration, spreading, and focal adhesions, but its role in tumor invasion and metastasis is still unclear [8]. The second most commonly mutated gene in GBM is *p53* [5]. In literature, conflicting data exist concerning the impact of *p53* status on the resistance to cancer therapy [9]. Recent data indicate that some of the most common mutant p53 proteins have, in addition to losing transcriptional function, acquired a gain of function in promoting tumor cell migration and metastasis [10, 11]. The effects of p53 on cell motility are largely mediated through the regulation of Rho signaling, thereby controlling actin cytoskeletal organization [12] and preventing filopodia formation, cell spreading, migration and invasion. Loss of p53 function increases the activities of RhoA and Rac through the activation of the PI3K/AKT/mTOR signaling (hereafter denoted as the PI3K pathway), and also causes overabundance of Cdc42-dependent filopodia formation. As a result, activation of this network promotes cell adhesion and migration [13].

In addition to PTEN and p53, the activated PI3K pathway in *PTEN*-mutated cells can increase the migration and invasion activity of cells through either inhibition of 4E-BP1 (*negative* regulator of migration) or activation of S6K1 (*positive* regulator of migration) proteins [14]. Therefore, inhibition of key proteins in this pathway, such as PI3K, AKT and/or mTOR, can be expected to reduce the migration of tumor cells.

Besides this, tumor cell migration depends on the overexpression of the heat shock protein 90 (Hsp90). As a molecular chaperone, Hsp90 promotes the post-translational maturation and maintains the stability of a large number of oncogenic client proteins, including those implicated in cell migration [15].

Given the major roles of PI3K/mTOR and Hsp90 in regulating tumor cell motility, we evaluated in this study whether their inhibitors, PI-103 and NVP-AUY922 (hereafter denoted as AUY922) have potential as anti-migratory agents in GBM. The novel synthetic molecule of the pyridofuropyrimidine class, PI-103, is a potent and selective inhibitor of class I PI3K [16], mTOR and DNA-PK, which exhibit therapeutic activity against a range of human tumor xenografts [17]. The second agent, NVP-AUY922, is an isoxazole resorcinol derivative with improved bioavailability, lower toxicity and high affinity for the NH_2_-terminal nucleotide-binding site of Hsp90 [18] along with beneficial pharmacological properties. It also exhibits strong antiproliferative effects against various tumor cell lines and primary tumors *in vitro* and *in vivo* at well-tolerated doses [19].

We analyzed the impact of either drug in combination with irradiation (IR) on the migration of two GBM cell lines differing in *PTEN* and *p53* status. The cell lines were analyzed for cell morphology, F-actin distribution, migratory behavior in wound healing and single-cell tracking assays, and expression of several marker proteins of the PI3K and ERK pathways. Proteins responsible for cell adhesion and actin cytoskeleton organization (FAK, RhoA, Cdc42, *etc*.) were also examined.

## RESULTS

### Migration patterns of GBM cells and effects of PI3K-, mTOR- and Hsp90-inhibition

**Figure 1:**
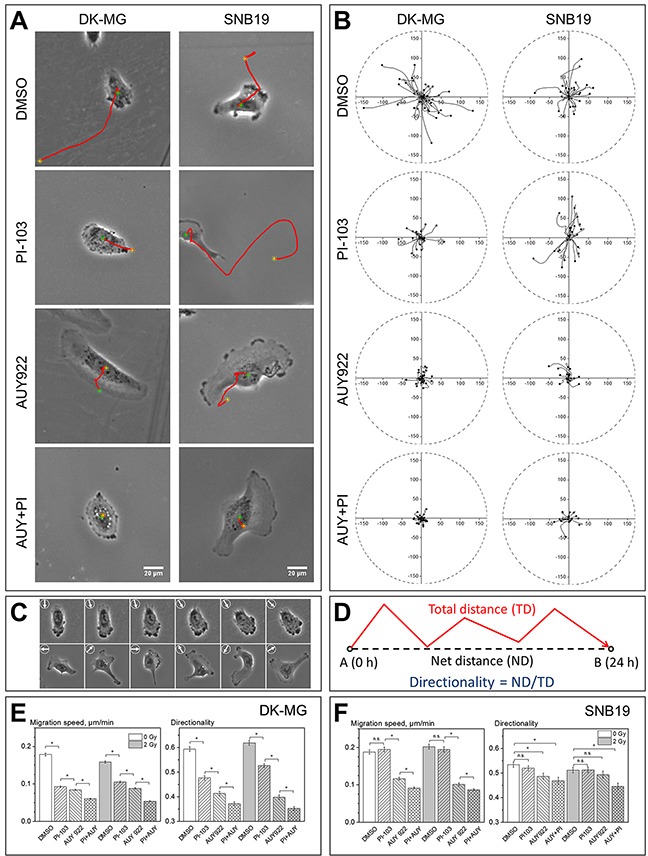
Impact of PI3K/mTOR and/or Hsp90 inhibition on the motility of DK-MG and SNB19 glioblastoma cell lines evaluated by single-cell tracking **(A)** Typical appearance and migratory behavior of control (DMSO) and drug-treated cells observed by phase-contrast microscopy. Red lines indicate cell migration paths over a period of 5 h. **(B)** Representative tracks of about 30 cells localized every 10 min for 5 hours, for each condition. The initial position of each cell was set to the origin (0,0) of coordinates. Black dots indicate the final cell locations at *time* = 5 h. The corresponding time lapse videos are available in Supplementary Videos 1 and 2. **(C)** Changes in cell morphology and direction of migration (arrows) for control DK-MG (upper row) and SNB19 (bottom row) cells. Images in **(C)** were taken in 100 min intervals over 500 min. **(D)** The terms *total* and *net distance*s, as well as the *directionality* of migration. Directionality of migration is defined as the net distance (ND, or displacement) from the starting position divided by the length of the total distance (TD). The results in **(E)** and **(F)** represent the mean values (± SE) of migration speed (*i.e*. *total* distance divided by time) and directionality **(D)** from at least 500 cells per condition. White and grey bars denote non-irradiated and irradiated (2 Gy) samples, respectively. * means *P* < 0.05. “n.s.” means “not significant”.

Using time-lapse video-microscopy we first examined the migration of individual DK-MG and SNB19 cells (Figure 1A, Supplementary Videos 1 and 2) using Time Lapse Analyzer. This software enables detailed analysis of individual cell tracks providing data on the migration speed and directionality (Figure 1D) of single cells.

As evident from the tracking diagrams of control samples shown in the upper row of Figure 1B, the final positions (black dots) of a large portion of DK-MG cells are more distant from the starting point than those of SNB19 cells. This finding suggests a higher directional persistence in the migration of control DK-MG cells as compared to a more random migration pattern of SNB19 cells. The latter conclusion is also supported by the statistical data shown in Figure 1E and 1F. Thus, although the migration speeds of control DK-MG and SNB19 cells are similar, the directionality index of DK-MG cells (Figure 1E) is significantly higher than that of control SNB19 cells (Figure 1F).

Besides different migration patterns, the two GBM lines also differ in the number of lamellipodia per cell (Figure 1C, Supplementary Videos 1 and 2). Thus, control DK-MG cells are strongly polarized in the direction of migration, exhibiting one large leading lamellipodium and a narrower trailing edge. Interestingly, at the leading edge of the lamellipodium, control DK-MG cells exhibit abundant transient blebs, visible in phase contrast microscopy as dark spherical expansions (Supplementary Video 1). Compared to control DK-MG cells, the random migration pattern of control SNB19 cells (Supplementary Video 2 and arrows in Figure 1C) might have resulted from uncoordinated activity of multiple lamellipodia formed by this cell line, exhibiting multipolar morphology and fast morphological changes (Figure 1C, Supplementary Video 2).

Treatment with either PI-103 or AUY922 decreased the migration speed of DK-MG cells by ∼50%, with respect to the DMSO-treated control (Figure 1E). Simultaneous treatment with PI-103 and AUY922 gave rise to a much stronger (67%) inhibition of DK-MG cell migration (Figure 1E). In sharp contrast to its strong effect on DK-MG cells, PI-103 alone did not affect the migration of SNB19 cells (Figure 1F). On the other hand, AUY922 given either alone or in combination with PI-103 significantly reduced the migration speed of non-irradiated SNB19 cells (Figure 1F), although to a lesser extent than in DK-MG cells (Figure 1E). In both cell lines, combination of PI-103 and AUY922 caused a stronger reduction in migration speed than either inhibitor alone (Figure 1E and 1F).

Irradiation with 2 Gy (grey bars in Figure 1E and 1F) caused little, if any, changes in the migration activities of single cells from both cell lines, as compared to the corresponding non-irradiated samples (white bars). Likewise, the effects of PI-103 and AUY922 on the migration of irradiated cells were similar to their effects on non-irradiated samples from both cell lines.

**Figure 2:**
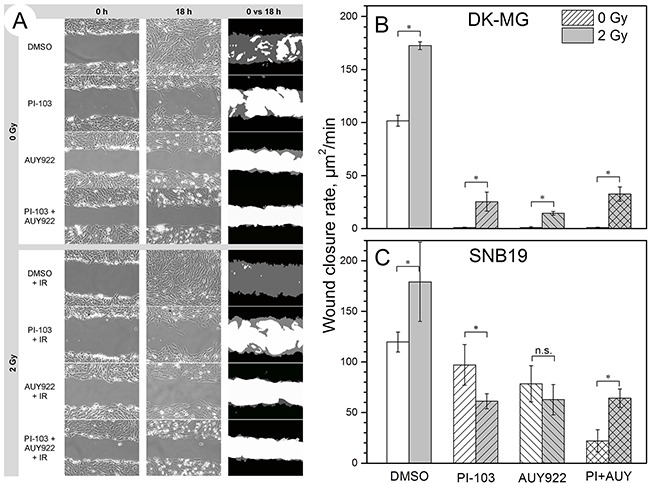
Effects of PI3K/mTOR and Hsp90 inhibition on the migration of non-irradiated and irradiated (2 Gy) cells studied by a wound healing assay Representative phase contrast images of DK-MG cells shown in the left and middle columns in **(A)** were acquired at 0 and 18 h intervals, respectively, after the scratch was made. White color in the RHS column of **(A)** represents the cell-free area, black and grey colors denote cell-covered areas at *time* = 0 and 18 h, respectively. The bar graphs in **(B)** and **(C)** summarize the impact of drug treatment on the wound closure rate, expressed in unit area per min [μm^2^/min], in DK-MG **(B)** and SNB19 **(C)** cell monolayers. Each bar represents the mean ± SE of at least three independent experiments made in quadruplicate.

In addition to single-cell tracking, we performed a wound healing assay. In agreement with the results of the single-cell tracking assay, drug-treated DK-MG cells closed the wound area much slower than control (*i.e*. drug-free and non-irradiated) cells (Figure 2A). The data of the wound healing test on both cell lines, are statistically summarized in Figure 2B and 2C. Treatment with PI-103, AUY922, or both agents combined almost completely inhibited migration of DK-MG cells into the wound (Figure 2A).

A further finding of the wound healing test was that SNB19 cells were less responsive to drug treatment than DK-MG cells (Figure 2C). Thus, compared to untreated controls, SNB19 cells treated with PI-103, AUY922, and both agents combined, exhibited, respectively, a 19, 34 and 82% decrease in the wound healing rate (Figure 2C), which is much less than the 100-fold inhibition observed in drug-treated DK-MG cells (Figure 2B). Interestingly, a single IR dose of 2 Gy accelerated the wound closure rate in control and drug-treated samples of both GBM lines (grey bars in Figures 2B and 2C).

### Effects of PI3K-, mTOR- and Hsp90-inhibition on the actin cytoskeleton

Cell migration is a complex process that involves coordinated changes in the F-actin cytoskeleton and focal adhesions [20]. In order to explain the different response of two GBM lines to a dual PI3K/mTOR inhibitor in terms of cell migration (Figures 1 and 2), we analyzed, via fluorescence microscopy, the drug-induced remodeling of actin filaments and focal adhesion complexes.

**Figure 3:**
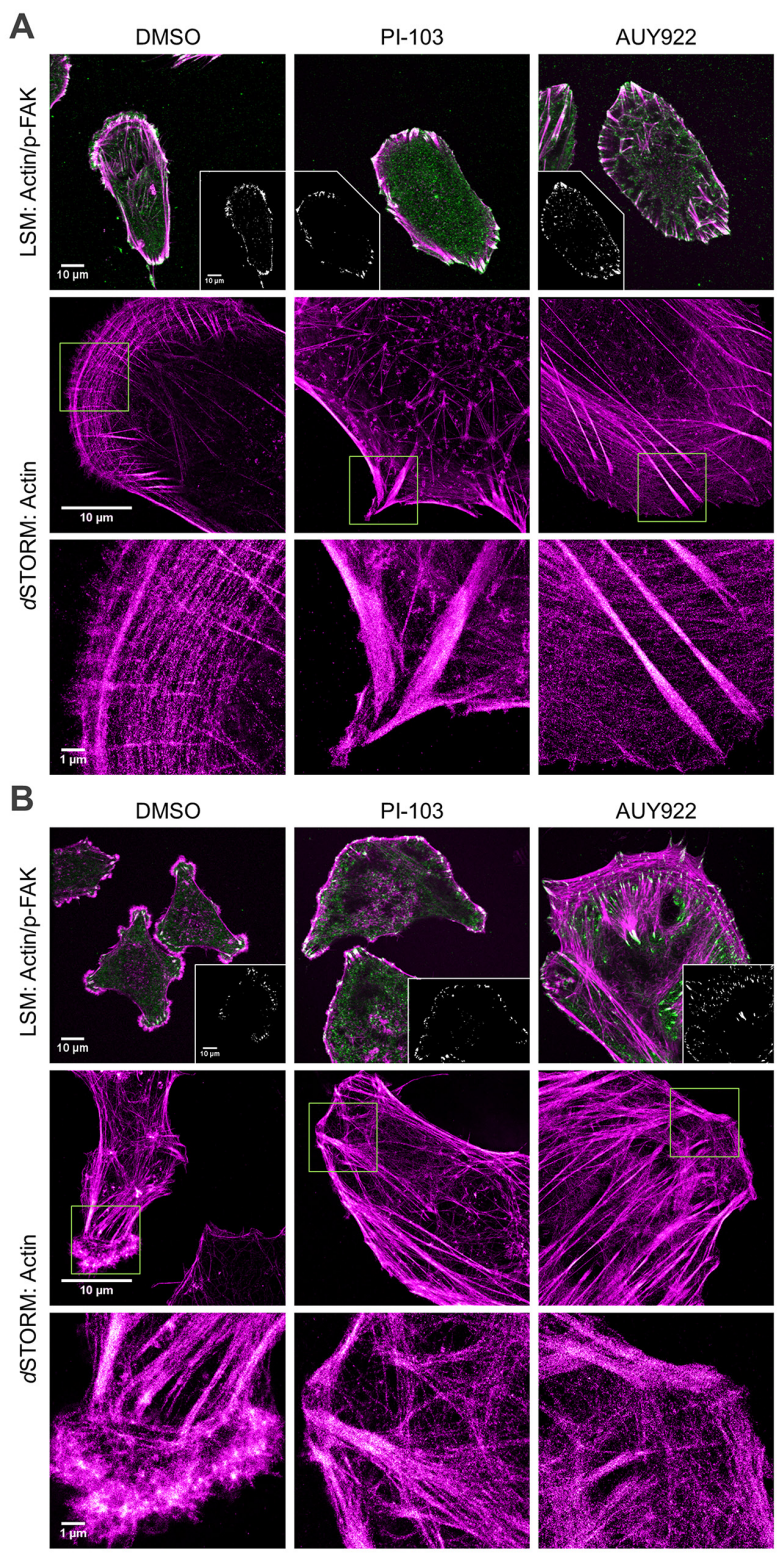
Effects of PI-103 and AUY922 on F-actin organization (magenta) and distribution of p-FAK(*Tyr397*) (green, upper row) in DK-MG **(A)** and SNB19 **(B)** cells. Images were acquired by confocal (LSM, upper row) and super-resolution (*d*STORM, middle and bottom rows) microscopy. Images show cell sections close to the basal plasma membrane in order to visualize focal adhesions and actin structure. Insets in the upper row illustrate co-localization of actin with p-FAK(*Tyr397*) (white area) at focal adhesion sites. The bottom row shows a magnification of the areas outlined by yellow squares in the corresponding *d*STORM images (middle row). F-actin filaments were visualized with Dy647-conjugated phalloidin. p-FAK(*Tyr397*) was immunostained with primary and secondary antibodies labeled with Alexa488 (*see* Supplementary Materials). Scale bars are 10 μm in the upper and middle rows and 1 μm in the bottom row, respectively.

Confocal laser scanning microscopy (LSM) demonstrated substantial differences between control DK-MG and SNB19 cells in the organization of the actin cytoskeleton and focal adhesions (Figure 3A and 3B). Combined staining of DK-MG cells for F-actin (magenta) and focal adhesion kinase phosphorylated at tyrosine 397 (p-FAK*Tyr397*, green) clearly shows their polarized shape (*i.e*. the front-rear polarity) along with different types of actin stress fibers, including dorsal fibers and transverse arcs, typical for the leading edge of the lamellipodium [21]. In addition to conventional LSM, the lamellipodial actin meshwork of control DK-MG cell was resolved in more detail by means of super-resolution imaging using direct stochastic optical reconstruction microscopy (*d*STORM) [22, 23], as shown in the two bottom pictures in the left-hand side (LHS) column of Figure 3A. While being evenly distributed through the cytosol of control DK-MG cells (top left image of Figure 3A), p-FAK is highly concentrated at the leading edge and, to a lesser extent, at the cell rear, where it co-localizes with F-actin within the focal adhesions (Figure 3A, inset in top left image).

In contrast to DK-MG cells, control SNB19 cells analyzed by LSM show multipolar morphology with several lamellipodia protruding from the cell body in all directions (top left image in Figure 3B), which agrees well with the phase-contrast images (Figure 1C). The tip of each lamellipodium shows diffuse actin staining along with p-FAK-enriched spots in the focal adhesions (Figure 3B, inset in top left image). *d*STORM images (Figure 3B, bottom left image) confirm diffuse actin staining, resulting most likely from the gel-like actin structure in the lamellipodial tips. Unlike unipolar DK-MG cells, multipolar SNB19 cells contain very few, if any, stress fibers and focal adhesions in the central regions.

Irrespective of the drug used, the most notable change in drug-treated DK-MG cells is the lack of a polarized cytoskeleton structure (Figure 3A) and of the transverse arcs seen in migrating control cells. Besides this, both LSM and *d*STORM reveal prominent actin stress fibers anchored to focal adhesions at the perimeter (Figure 3A) of PI-103-treated DK-MG cells. The central region of these cells contains neither ventral stress fibers nor focal adhesions detectable by LSM. However, taking advantage of super-resolution *d*STORM we found tiny, randomly oriented actin fibers with a diameter of 70-200 nm (*i.e*. beyond the resolution limits of LSM) close to the ventral plasma membrane. AUY922-treated DK-MG cells display abundant radial stress fibers terminated with focal adhesions at the cell periphery (Figure 3A). Although PI-103 did not affect the migration of SNB19 cells in the single-cell tracking test (Figure 1F) or exhibited at best modest efficacy in the wound healing test (Figure 2C), it caused a reorganization of actin filaments, most notably the loss of multiple lamellipodia, seemingly due to them merging into one large lamellipodium. The actin-rich leading edge of this lamellipodium contains abundant focal adhesions, whereas the cell rear is largely devoid of F-actin and focal adhesions.

Along with strongly impeded migration, SNB19 cells treated with AUY922 exhibit a marked enlargement of the projected cell area, numerous actin stress fibers and focal adhesions in the central regions, as well as dense actin staining and focal adhesions along the entire cell border (Figure 3B), which is consistent with our previous results obtained by epifluorescence microscopy [24]. In both cell lines, a single radiation dose of 2 Gy applied alone or in combination with either inhibitor did not cause any significant changes in the actin cytoskeleton, as compared to the corresponding non-irradiated samples (data not shown).

### Effects of PI3K-, mTOR- and Hsp90-inhibition on marker protein expression

**Figure 4:**
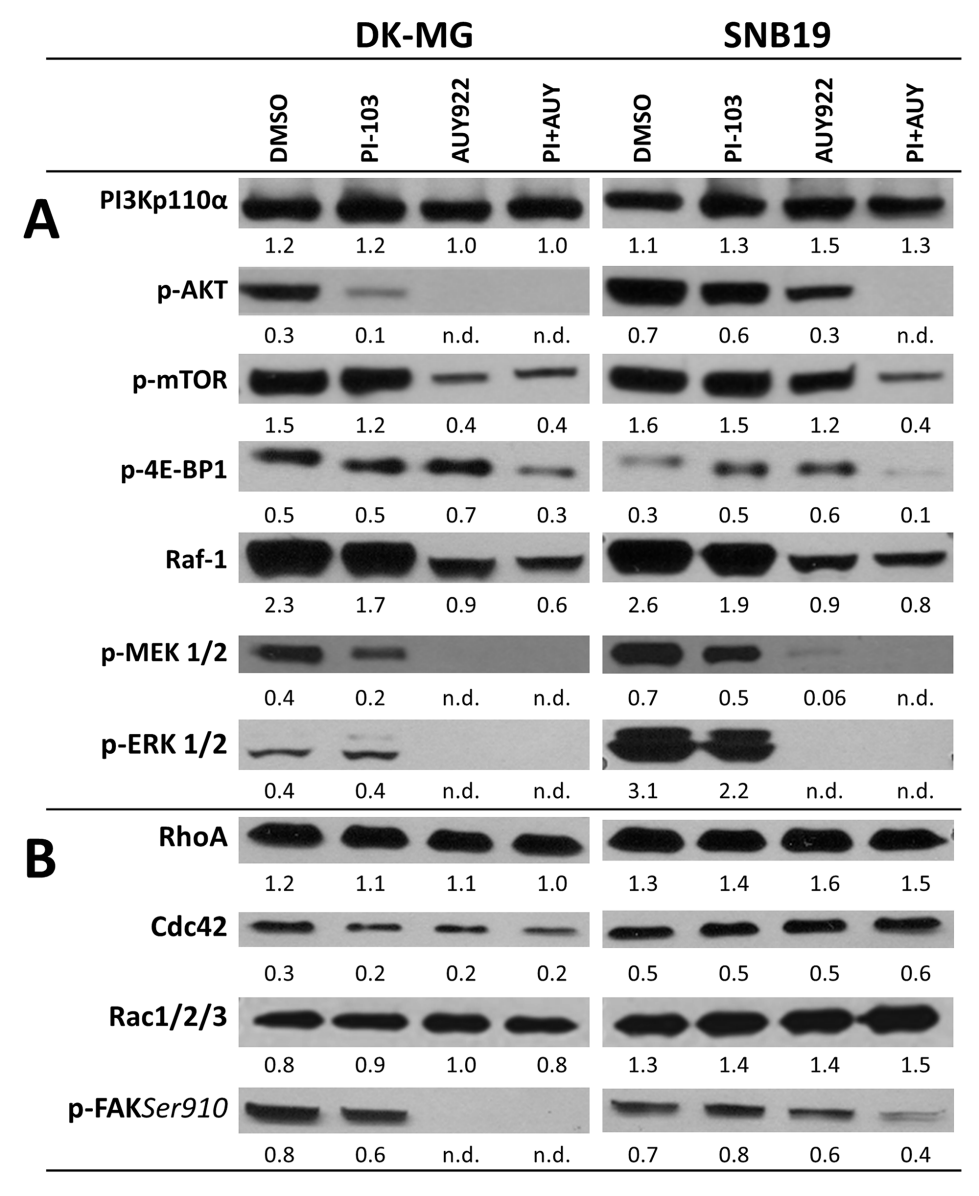
Representative Western blots of several marker proteins of PI3K- and ERK-pathways **(A)**, the Rho GTPases (RhoA, Cdc42 and Rac 1/2/3) and p-FAK(*Ser910*) **(B)** in DK-MG and SNB19 cells treated with DMSO (control) or the indicated inhibitors for 24 h. Each protein band was normalized to the intensity of β-actin used as loading control. The protein/β-actin ratios are denoted by the numbers. The experiments were repeated at least three times.

The data presented in Figures 1–3 demonstrate the ability of the PI3K/mTOR/Hsp90 inhibitors to modify the morphology of GBM cells and, most notably, to inhibit their migration, in a cell line-specific manner. To gain an insight into the mechanisms responsible for the observed differences between the two cell lines, we examined by Western blot the expression of two groups of marker proteins. The first group of proteins (Figure 4A) includes targets of the dual inhibitor PI-103, *i.e*. PI3K and mTOR, along with p-AKT and p-4E-BP1. Because of the known crosstalk between PI3K and ERK pathways [25], proteins of the ERK signaling pathway (Raf-1, p-MEK1/2 and p-ERK1/2) were also tested. The ERK pathway, which is frequently mutated in cancer cells [26], transmits signals from surface receptors to promote survival, proliferation and migration [27].

The second group of proteins (Figure 4B) includes the Rho GTPases, namely RhoA, Cdc42, and Rac1/2/3, which require phosphorylation by pro-survival signaling through AKT and ERK2 [28]. The members of the Rho GTPase family regulate intracellular actin dynamics, cytoskeleton remodeling, cell polarity and lamellipodium extension [29–32]. In addition, we detected p-FAK(*Ser910*). Representative Western blots of the indicated proteins are shown in Figure 4 for control (DMSO) and drug-treated (24 h) DK-MG and SNB19 (LHS and right-hand (RHS) columns of the figure, respectively) cells. The data on short-term (3 h) treatment are shown in Supplementary Figure 1.

As seen in Figure 4A, control DK-MG and SNB19 cells contain comparable amounts of PI3K, p-mTOR and Raf-1. At the same time, expression of p-AKT, p-MEK1/2 and p-ERK1/2 in control SNB19 cells is much higher than in control DK-MG cells. The increased level of p-AKT (Figure 4A) in *PTEN*-mutated SNB19 cells (Supplementary Figure 2) is consistent with the findings that PTEN loss can lead to compensatory activation of the PI3K pathway [33]. The amount of p-4E-BP1 in DK-MG cells exceeds that of SNB19 cells. The level of p-FAK(*Ser910*) in control SNB19 cells is 25% lower than in control DK-MG cells (Figure 4B). At the same time, SNB19 cells show higher (by 20-50%) expression levels of the Rho GTPases (RhoA, Cdc42 and Rac1/2/3), which might be associated with the *p53* mutation in SNB19 cells, since loss of p53 function activates RhoA and Rac, and also causes overabundance of Cdc42-dependent filopodia formation [13].

Prolonged incubation with PI-103 caused a significant decrease in p-AKT, p-MEK1/2, Raf-1 and p-mTOR in DK-MG cells, but it had little, if any, impact on these proteins in SNB19 cells. Interestingly, PI-103 increased the expression of the phosphorylated form of 4E-BP1 in SNB19 cells which can result in an activation of overall translation and migration, whereas its content was almost unchanged in PI-103-treated DK-MG cells. Besides this, PI-103 strongly decreased the expression of Cdc42 and p-FAK(*Ser910*) in DK-MG cells, without significantly affecting RhoA and Rac1/2/3. In PI-103-treated SNB19 cells, there was a slight increase in Rho GTPase and p-FAK(*Ser910*) levels, with respect to DMSO-treated control. As expected, treatment with AUY922 strongly increased the expression of Hsp90 (Supplementary Figure 3) but decreased the expression of Hsp90 client proteins [15], *e.g*. p-AKT, p-MEK1/2, and p-ERK1/2, in both cell lines. Interestingly, the expression of p-AKT was almost completely abolished in AUY922-treated DK-MG cells whereas it was decreased only by a factor of ∼2 in the respective SNB19 cell samples. This may be due to the large difference in p-AKT expression levels between the control samples of the two cell lines. Determination of the non-phosphorylated forms of the marker proteins (Supplementary Figures 4 and 5) revealed that their expression was less affected by either inhibitor as compared to the respective phosphorylated forms of the proteins (Figure 4).

## DISCUSSION

In this study, we tested the effects of targeting the PI3K pathway using the dual PI3K/mTOR inhibitor PI-103 with the intention of preventing the migration of two GBM cell lines differing in *p53* and *PTEN* status [34] as well as in their invasive capacities [35]. As an alternative approach, we evaluated the anti-migratory potential of the Hsp90 inhibitor AUY922. Briefly, we found that (*a*) the two GBM lines exhibited very different migratory patterns, *i.e*. directionally persistent *vs*. random movement; (*b*) inhibition of PI3K/mTOR greatly reduced migration of the less invasive DK-MG cells (*PTEN* wt, *p53* wt), without affecting migration of the highly invasive SNB19 cells (*PTEN* mut, *p53* mut); (*c*) in contrast, the Hsp90 inhibitor AUY922 exerted in both cell lines strong anti-migratory effects, which were further enhanced by the addition of PI-103 (Figures 1 and 2).

The most striking difference between the two GBM lines, revealed by single-cell tracking, is that DK-MG cells move persistently in one direction, whereas SNB19 cells exhibit a more random migration (Supplementary Videos 1 and 2). A further difference is that only DK-MG cells, but not SNB19 cells, form abundant membrane blebs at the leading edge of the lamellipodium (Supplementary Video 1). Such membrane blebbing has also been observed in various other cell types during migration, both *in vitro* and *in vivo* [36].

An interesting finding is the significant increase in the wound closure rates observed in both cell lines after exposure to IR (Figure 2). Radiation-induced acceleration of GBM cell migration has already been reported [37]. This effect could be due to IR-induced release of exosomes, which has been shown to enhance GBM cell migration [38]. The abundance of exosomes in multicellular systems (such as a confluent cell monolayer) would explain why IR led to increased cell migration in the wound healing assay (Figure 2), but not in the single-cell tracking test (Figure 1).

**Figure 5:**
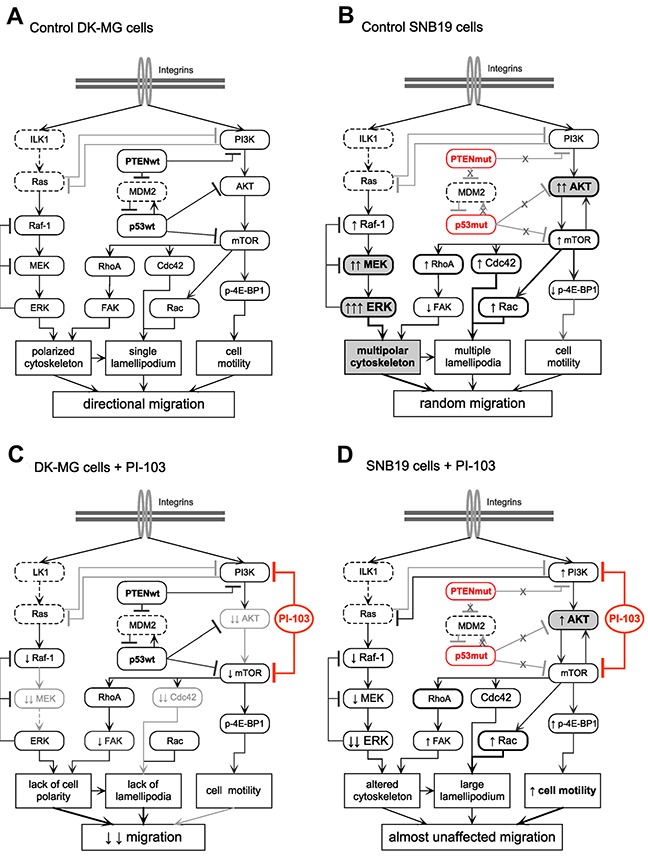
Simplified diagrams of putative signaling pathways responsible for the different migration patterns (*i.e*. directionally persistent *vs*. random) of control DK-MG **(A)** and SNB19 **(B)** cells, as well as for the cell line-specific effect of the PI3K/mTOR inhibitor PI-103 on cell migration **(C)** and **(D)**. In contrast to DK-MG cells, the PTEN-mutated SNB19 cells express constitutively activated PI3K- and ERK-pathways which favor migration and invasion of these cells. Besides this, the protein p53 is mutated in SNB19 cells, which might also influence their migration phenotype, *e.g*. increased expression of RhoA, Cdc42 and Rac1/2/3. Incubation of SNB19 cells with PI-103 for 24 h **(D)** leads to a rebound activation of the PI3K/AKT/mTOR pathway, most likely due to inhibition of the negative feedback loop mediated by ribosomal protein S6, resulting in mostly unaltered motility. In contrast, in PI-103-treated DK-MG cells, which lost their motility, the expression of p-AKT remained almost undetectable. The diagram is based on the data presented in Figures 1–4 and Supplementary Videos 1 and 2, and also includes data published elsewhere [25, 26, 29, 30]. Non-determined proteins are marked with dashed lines. The symbols ↑ and ↓ denote, respectively, increased and decreased protein expression levels compared to corresponding control cells **(A, B)**, as detected by Western blot analysis shown in Figure 4. (For detail, *see* the Discussion section.)

The different migration patterns of the two cell lines apparently result from the cell line-specific expression profiles of relevant proteins (Figure 4), which in turn might be associated with the *PTEN* (and possibly *p53*) mutation status of these cell lines. The observed differences in cell migration patterns between the two cell lines are explained by a simplified model presented in Figures 5A and 5B.

In general, our Western blot detections (Figure 4) showed higher activities of the key components of the PI3K and ERK pathways in *PTEN*-mutated SNB19 cells, as compared to the *PTEN* wild-type DK-MG cells. Both PI3K and ERK pathways include critical factors not only for enhanced proliferation and survival, but also for the promotion of migratory and invasion machineries [31, 39–41].

In addition to *PTEN*, cell motility is controlled by *p53* [13], present as wild-type in DK-MG, but mutated in SNB19 cells. A deficiency in *p53* has been shown to be implicated in the development of highly aggressive tumors [42]. As reviewed elsewhere [13], loss of *p53* function promotes (via activation of PI3K pathway) upregulation of Rho GTPases, which is consistent with the results reported here showing higher Rho GTPase (RhoA, Cdc42, and Rac) levels in *p53*-mutated SNB19 cells as compared to p53 wild-type DK-MG cells (Figure 4B).

Along with the differences between control cells of the two GBM lines, the most important finding of this study, confirmed by two independent migration assays (Figures 1 and 2), is that PI3K/mTOR inhibition by PI-103 exerted a strong anti-migratory effect only on DK-MG but not on SNB19 cells. The potential of PI-103 as an inhibitor of cell migration has been tested by Ströbele et al. [43], who report that PI-103 reduces cell motility in two established and three primary GBM cell lines.

Cell line-specific effects of PI-103 (Figures 1 and 2) are also evident on the protein expression level (Figure 4). Particularly, PI-103 has a strong inhibitory effect on the expression of p-AKT, p-MEK1/2, p-FAK(*Ser910*) and Cdc42 only in DK-MG cells. Based on our results of the migration tests (Figures 1 and 2), fluorescence microscopy of the cytoskeleton (Figure 3A and 3B) and Western blot analysis (Figure 4), we propose a simplified model explaining the anti-migratory effect of PI-103 on DK-MG cells and the lack of effect on SNB19 cells (Figure 5C and 5D).

A possible reason for the failure of PI-103 to inhibit migration of SNB19 cells might be rebound activation of AKT in this cell line upon 24-h drug exposure (Figures 4A and 5D), which occurred after a transient p-AKT depletion observed in cells treated with PI-103 for 3 h (Supplementary Figure 1). In contrast, PI-103-treated DK-MG cells remained almost depleted of p-AKT (Figures 4A and 5C) during the whole incubation period. We hypothesize that reactivated AKT and the signaling events downstream of it may contribute (via a yet unknown mechanism) to the unaffected motility of PI-103-treated SNB19 cells. One explanation is that activated AKT is known to promote cell motility downstream of Rac/Cdc42 GTPases thereby modulating the cytoskeleton in growth factor-stimulated cells and in invasive PTEN-deficient cells [44], such as the SNB19 cells studied here.

In addition, given that both activated FAK and Cdc42 are important for maintaining a polarized cytoskeleton and for forming membrane protrusions [45], the observed reduction of p-FAK(*Ser910*) and Cdc42 in PI-103-treated DK-MG cells might be responsible for the loss of cell polarity and inability to form lamellipodia, resulting in an overall inhibition of DK-MG cell migration (Figure 5C).

Judging from the results of both migration assays (Figures 1 and 2), the Hsp90 inhibitor AUY922 seems to be a more potent anti-migratory agent in GBM cells than the PI3K/mTOR inhibitor PI-103. The advantage of AUY922 is that, unlike PI-103 which had no effect on SNB19 cell migration, AUY922 strongly impeded the migration of both tested cell lines (Figures 1 and 2), independent of their mutation status in *PTEN* and *p53*. As outlined in Supplementary Figure 6, the inhibition of cell migration by AUY922 seems to be mediated through various Hsp90 client proteins [15] such as AKT and MEK, which belong to different signaling pathways involved in cell motility and migration. On the cellular level, the AUY922-mediated alterations in the signaling pathways of SNB19 cells (Figure 4) were associated with massive changes in cell morphology and remodeling of the cytoskeleton. Particularly, in agreement with our results reported earlier [24], AUY922-treated SNB19 cells did not form multiple lamellipodia typical for control cells.

In conclusion, our study provides a proof-of-concept that dual inhibition of PI3K/mTOR is a promising therapeutic strategy for preventing migration of GBM cells. In case of migrating cells that are not responsive to prolonged PI3K/mTOR inhibition due to an aberrant reactivation of the PI3K pathway, the therapeutic window needs to be carefully defined, or a combination of AKT and Hsp90 inhibitors should be considered.

## MATERIALS AND METHODS

### Cell culture

Human glioblastoma (GBM) cell lines, DK-MG and SNB19, were obtained from DSMZ (Braunschweig, Germany) and routinely cultured in complete growth medium (CGM) under standard conditions (5% CO_2_, 37°C). CGM contained Dulbecco's modified Eagle's medium (DMEM, Sigma, Deisenhofen, Germany) supplemented with 10% fetal bovine serum. The population doubling times for DK-MG and SNB19 cells were found to be about 48 and 25 h, respectively. Both cell lines were authenticated by the supplier, used at low passage number (<15) and were mycoplasma free (MycoAlert; Lonza, Rockland, ME).

### Drug treatment

Both drugs were obtained from Selleckchem (Absource Diagnostics GmbH, Munich, Germany). The drugs were freshly diluted from frozen aliquots in DMSO stored at -20°C. PI-103 (2 μM, [46]) and NVP-AUY922 (50 nM) were added 3 h prior to exposure to ionizing radiation (IR) and remained in CGM up to 24 h post-IR. Cells treated in parallel with respective amounts of DMSO served as controls. In order to allow the cells to reach confluency and to avoid cell detachment in the wound healing test, the formerly used NVP-AUY922 concentration of 200 nM [47] was decreased to 50 nM. This concentration was used throughout the whole study.

### Antibodies

The primary and secondary antibodies used in this study are specified in Supplementary Information.

### X-ray irradiation

Irradiation was performed at room temperature using a 6 MV Siemens linear accelerator (Siemens, Concord, CA) at a dose rate of 2 Gy/min. After irradiation, cells were kept in CGM for the indicated time until harvest.

### Time-lapse phase-contrast microscopy

A Nikon BioStation IM-Q (Nikon, Melville, NY), which includes a cell incubator (37°C, 5% CO_2_), a motorized inverted microscope and a CCD camera, was used to image live GBM cells over time by phase contrast microscopy. Prior to single-cell tracking experiments, about 10^4^ cells were plated into a Petri dish (diameter 35 mm) containing 2 ml CGM. In each experiment, time-lapse images of several fields of view (706×530 μm) were acquired every 10 min over a 24-h period, using a ×10 phase contrast objective.

### Cell tracking and migration data analysis

Cell tracking was performed using the software Time Lapse Analyzer (TLA; University of Ulm, Germany), which enables an automated cell identification and tracking procedure suitable for the evaluation of video sequences of unstained cells. The image pre-processing, segmentation, *i.e*. the separation of the objects of interest (cells) from the background, and cell tracking were performed as described elsewhere [48] with minor modifications. Briefly, the acquired digital images were processed with TLA software by using a combination of entropic and median filtering algorithms, which yielded two sets of binary images. By applying an AND gate to the data sets, individual cells were discriminated from the background and the coordinates of the centroids of cell nuclei were computed. Using this data, the cell trajectories were reconstructed using a modified nearest-neighbor algorithm. Typically, 20 to 40 individual cells were tracked during each 24-h migration experiment. At least three experiments were conducted for each condition examined in the present study. From the acquired migration paths, cell migration speed and directionality of migration were calculated for each individual cell. Migration speed was defined as the total distance (TD) a cell travelled divided by the total time. Directionality (or persistence) of migration was defined as the net distance (ND, or displacement) from the starting position divided by the length of the total distance (TD), *e.g*. directionality = 1.0 for migration in a straight line, directionality = 0.0 for circular migration.

### Wound healing assay

Wound healing in the presence or absence of inhibitors, with or without IR treatment, was analyzed directly and 18 h after scratching as previously described [24].

### Fluorescence staining of F-actin and p-FAK(*Tyr397*)

Cells were cultured on glass slides to sub*-*confluency, permeabilized and fixated as described previously [49]. Staining of F-actin was performed with a phalloidin-Dy647-conjugate. Immunostaining of p-FAK(*Tyr397*) was performed by incubating samples with a primary and respective secondary antibody (*see* Supplementary Materials), each for 2 h at room temperature. Confocal fluorescence images were acquired with a Zeiss LSM 700 microscope.

### *d*STORM (direct stochastic optical reconstruction microscopy)

The experimental setup for *d*STORM was described previously in detail [23]. The main advantage of direct stochastic optical reconstruction microscopy (*d*STORM, [23]) over the conventional LSM is its superior spatial resolution of about 20 nm whereas in a standard LSM the resolution is limited to ∼250 nm due to light diffraction. *d*STORM uses photoswitchable fluorescent dyes, which can be transferred to a reversible dark state with a lifetime ranging between ∼100 ms and a few seconds. A sparse subset of fluorophores is then spontaneously (stochastically) reactivated, allowing their precise localization. Photoswitching is based on thiol-mediated reduction of fluorescent dyes to a non-fluorescent dark state after excitation. Repetitive cycles of activation, localization and deactivation enable a temporal separation of fluorophores from which a spatially super-resolved image can be reconstructed. Prior to the measurements, 5×10^4^ cells were seeded on 8-well II chambered cover glasses (Sarstaedt, Nümbrecht, Germany). For data processing and image reconstruction, the open access software for single-molecule-based localization microscopy *rapid*STORM 3.2 was used as previously described [50].

### Western blot

For immunoblot analysis, whole-cell lysates were prepared 3 h and 24 h after addition of the drugs according to standard procedures. Samples equivalent to 20-40 μg of protein were separated using 4-12% SDS-polyacrylamide pre-cast gels (Invitrogen, Karlsruhe, Germany) and transferred to nitrocellulose membranes, as described previously [46].

### Statististics

Data are presented as mean ± SE, unless otherwise noted. A Student's unpaired *t*-test was performed when statistical comparisons were made between two sets of data. The threshold of statistical significance was set at *P*-value of <0.05. Statistics was performed with Origin 8.5 (Microcal, Northampton, MA).

## SUPPLEMENTARY FIGURES AND VIDEOS






