# Supplementary material for: Migration pattern, actin cytoskeleton organization and response to PI3K-, mTOR-, and Hsp90-inhibition of glioblastoma cells with different invasive capacities
Source: Oncotarget. 2017 Apr 5;8(28):45298–310. doi: 10.18632/oncotarget.16847 (PMC5542187; doi:10.18632/oncotarget.16847)
Supplement: Supplementary file 1 [file oncotarget-08-45298-s001.pdf]

Migration pattern, actin cytoskeleton organization and response to PI3K-, mTOR-, and Hsp90-inhibition of glioblastoma cells with different invasive capacities

SUPPLEMENTARY MATERIALS

MATERIALS AND METHODS

Supplementary Information

The primary antibodies used were: rabbit monoclonal anti-PI3K p110α, mouse monoclonal anti-phospho-AKT (Ser473), rabbit monoclonal anti-phospho-mTOR (Ser2448), mouse monoclonal anti-phospho-4E-BP1, rabbit monoclonal anti-phospho-MEK1/2 (Ser217/221), rabbit monoclonal anti-phospho-p44/42 MAPK (ERK1/2) (Thr202/Tyr204), rabbit monoclonal anti-Cdc42, rabbit polyclonal anti-Rac1/2/3, rabbit polyclonal anti-phospho-Rac1/cdc42(Ser71), rabbit monoclonal anti-RhoA (all from Cell Signaling Technologies Inc., Danvers, MA), rabbit polyclonal anti-FAK(pS^{910}), rabbit polyclonal anti-Raf-1, mouse monoclonal anti-β-actin (Sigma, Deisenhofen, Germany). Secondary species-specific antibodies for western blot were labelled with horseradish-peroxidase (DAKO, Hamburg, Germany).

F-actin and FAK staining

F-actin was stained using DY-647P1-Phalloidin (Dyomics, Jena, Germany) according to the manufacturer's instructions. p-FAK(*Tyr397*) was stained using primary rabbit polyclonal anti-FAK(pY^{397}) (Santa Cruz Biotechnology, Dallas, USA) and secondary goat anti-rabbit polyclonal IgG Alexa 488 (Life Technologies, Darmstadt, Germany) antibodies.

REFERENCE

1. Amiri A, Noei F, Feroz T, Lee JM. Geldanamycin anisimycins activate Rho and stimulate Rho- and ROCK-dependent actin stress fiber formation. Mol Cancer Res. 2007; 5: 933–942.

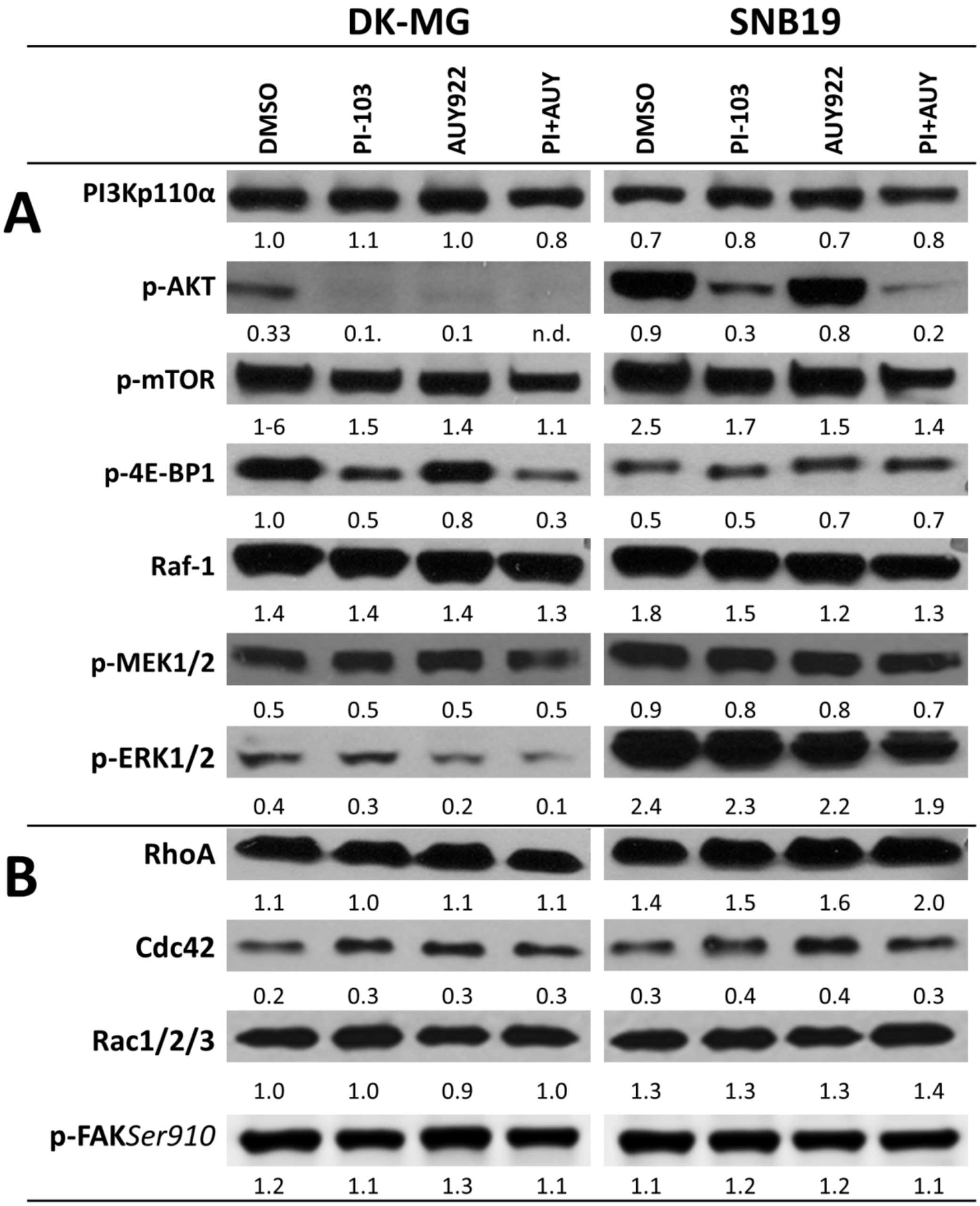

Supplementary Figure 1: Representative Western blots of several marker proteins of the PI3K- and ERK-pathways **(A)**, the Rho GTPases (RhoA, Cdc42 and Rac1/2/3) and p-FAKSer910 **(B)** in DK-MG and SNB19 cells treated with DMSO (control) or the indicated inhibitors for 3 h. Each protein band was normalized to the intensity of β-actin used as loading control. The protein/β-actin ratios are denoted by the numbers. The experiments were repeated three times. For details, *see* legend to Figure 4.

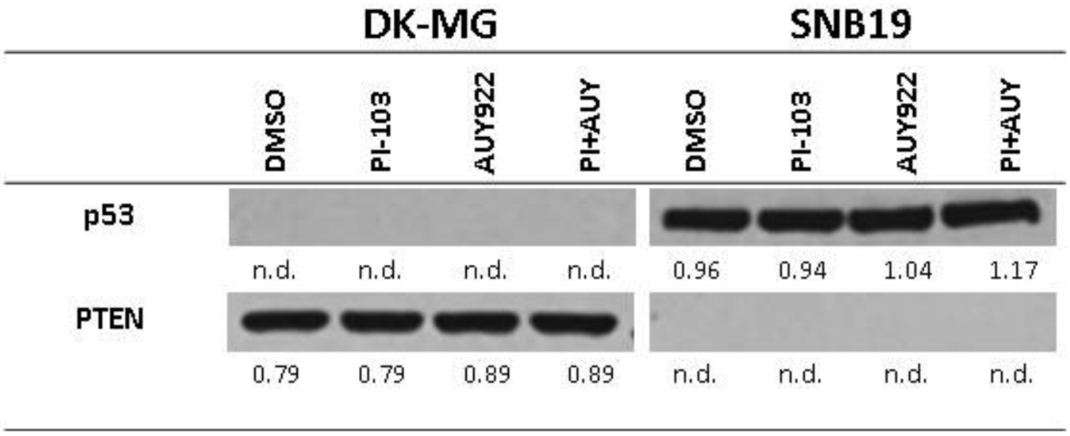

Supplementary Figure 2: Representative Western blot showing the expression of p53 and PTEN proteins in DK-MG (*p53* wt, *PTEN* wt) and SNB19 (*p53* mut, *PTEN* mut) cells treated with DMSO (control) or the indicated inhibitors for 3 h. Each protein band was normalized to the intensity of β-actin used as loading control. The protein/β-actin ratios are denoted by the numbers. The experiments were repeated three times.

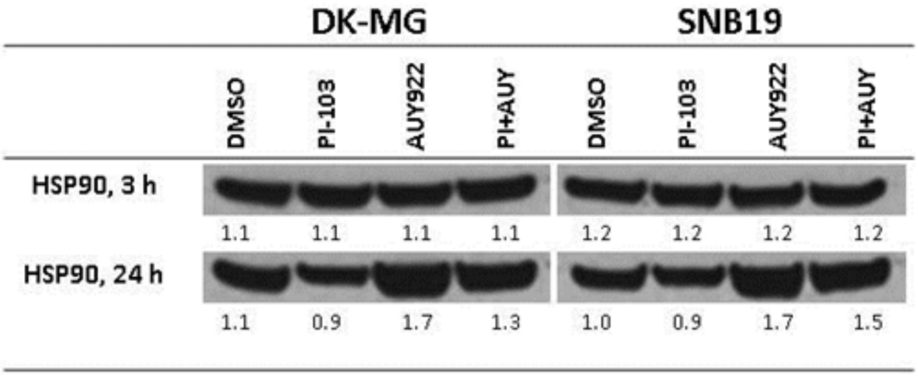

Supplementary Figure 3: Representative Western blot showing the expression of Hsp90 protein in DK-MG and SNB19 cells treated with DMSO (control) or the indicated inhibitors for 3 or 24 h. Each protein band was normalized to the intensity of β-actin used as loading control. The protein/β-actin ratios are denoted by the numbers. The experiments were repeated three times.

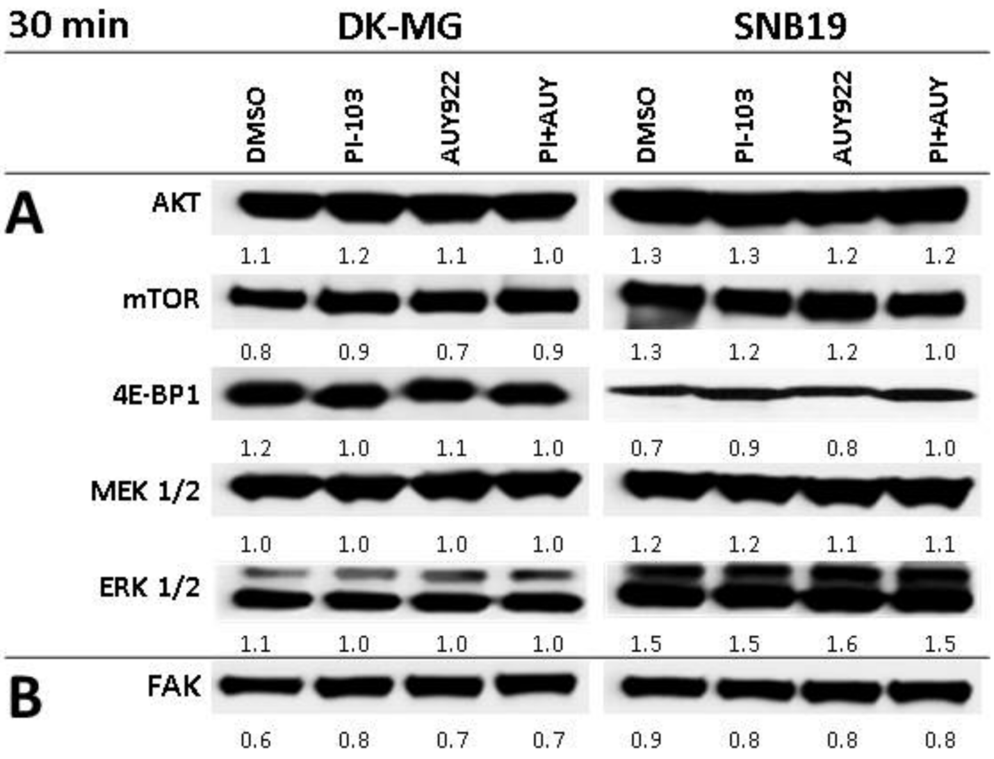

Supplementary Figure 4: Representative Western blots of several marker proteins of the PI3K- and ERK-pathways **(A)**, and the FAK **(B)** in DK-MG and SNB19 cells treated with DMSO (control) or the indicated inhibitors for 3 h. Each protein band was normalized to the intensity of β-actin used as loading control. The protein/β-actin ratios are denoted by the numbers. The experiments were repeated three times. For details, *see* legend to Figure 4.

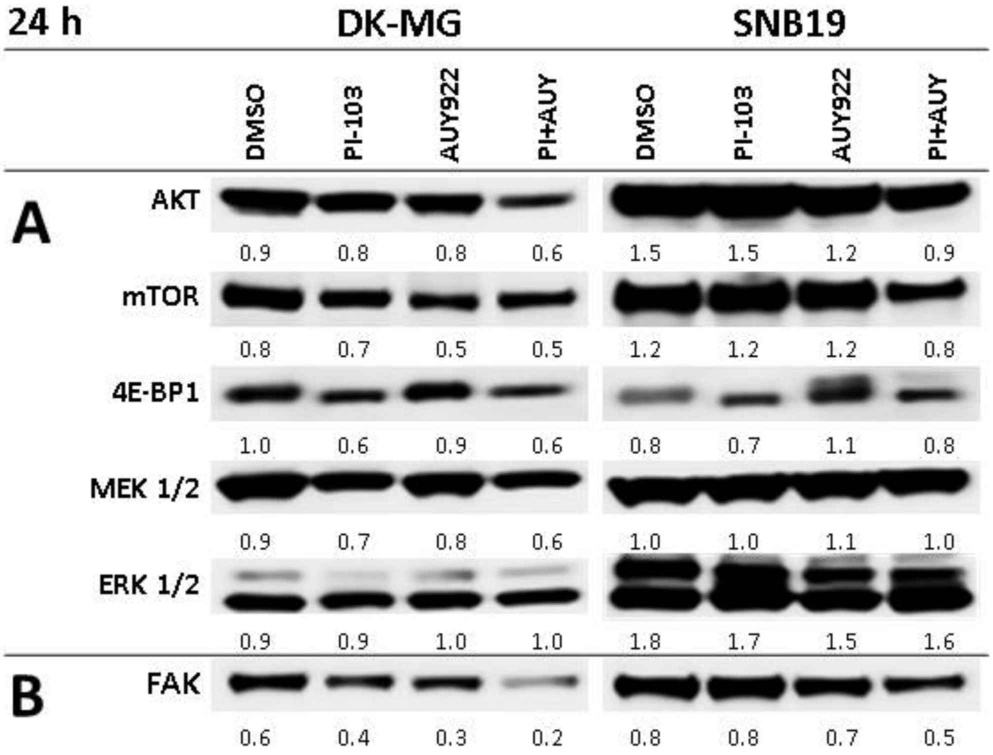

Supplementary Figure 5: Representative Western blots of several marker proteins of the PI3K- and ERK-pathways **(A)**, and the FAK **(B)** in DK-MG and SNB19 cells treated with DMSO (control) or the indicated inhibitors for 24 h. Each protein band was normalized to the intensity of β-actin used as loading control. The protein/β-actin ratios are denoted by the numbers. The experiments were repeated three times. For details, *see* legend to Figure 4.

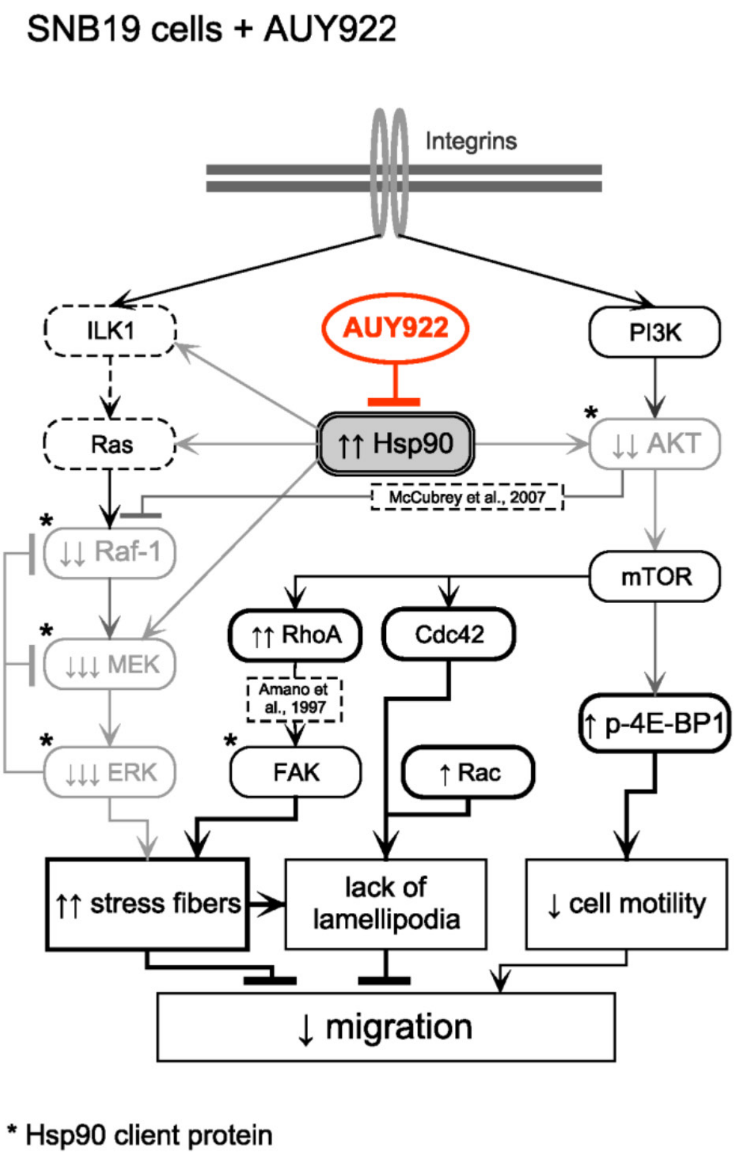

Supplementary Figure 6: Simplified diagram of putative signaling pathways relevant for the inhibition of migration of SNB19 cells treated with the Hsp90 inhibitor AUY922. As a result of Hsp90 inhibition, the expression of its client proteins ILK-1, Raf-1, MEK, AKT and FAK decreased. Raf-1, MEK and ERK are components of the ERK signaling pathway, which plays a major role in cell migration and many other cellular activities [27]. Depletion of ILK-1, Raf-1 and MEK might have caused reduced expression of p-ERK, thus leading to alterations in actin structure and inhibition of cell migration. A further Hsp90 client protein tested here was AKT, whose activation normally results in inhibition of Raf-1 and downstream MEK/ERK [25]. This cross-talk between the PI3K- and ERK-pathways can cause an additional depletion of p-ERK, which can further impede cell migration. In addition, RhoA, which regulates actin polymerization leading to the formation of stress fibers, was found to be up-regulated in SNB19 cells treated with AUY922. Rho activation normally induces focal adhesions via FAK stimulation [Amiri et al, 2007]. However, as an Hsp90 client protein, FAK (not shown) and its phosphorylated form were strongly depleted in DK-MG but not in SNB19 cells treated with the Hsp90 inhibitor (Figure 4). RhoA activation und the development of stress fibers lead to the observed changes in cell spreading, migration and adhesion. The proposed diagram is based on the data presented in Figures 1-4 and Supplementary Figure 3. The diagram also includes published data [25, 26, 29, 30]. (For detail, *see* the Discussion section).

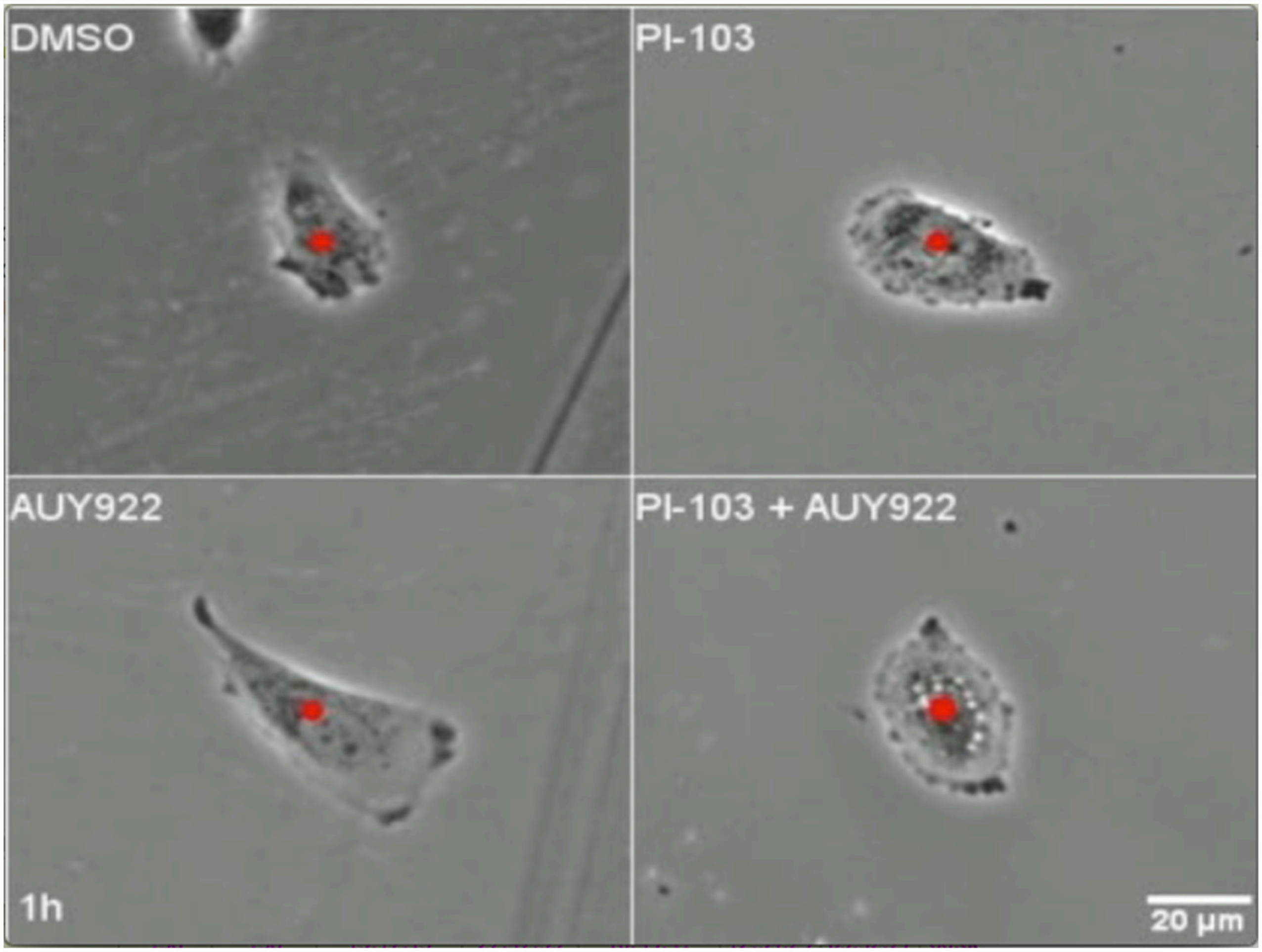

Supplementary Video 1: DK-MG cells were imaged every 10 min by time-lapse microscopy using a Nikon BioStation IM-Q (Nikon, Melville, NY). Prior to single-cell tracking experiments, about 10^4 cells were plated into a Petri dish (diameter 35 mm) containing 2 ml complete growth medium. In each experiment, time-lapse images were acquired over an 18-h period, using a ×10 phase contrast objective. Control DMSO-treated cells are shown in the top left movie. Drug-treated cells were pretreated 3 h before recording either with PI-103 (top right movie), AUY922 (bottom left movie) or a combination of both inhibitors (bottom right movie). The movies are played back at a rate of 15 frames per second (speed up 9000× real time).

See Supplementary Video 1

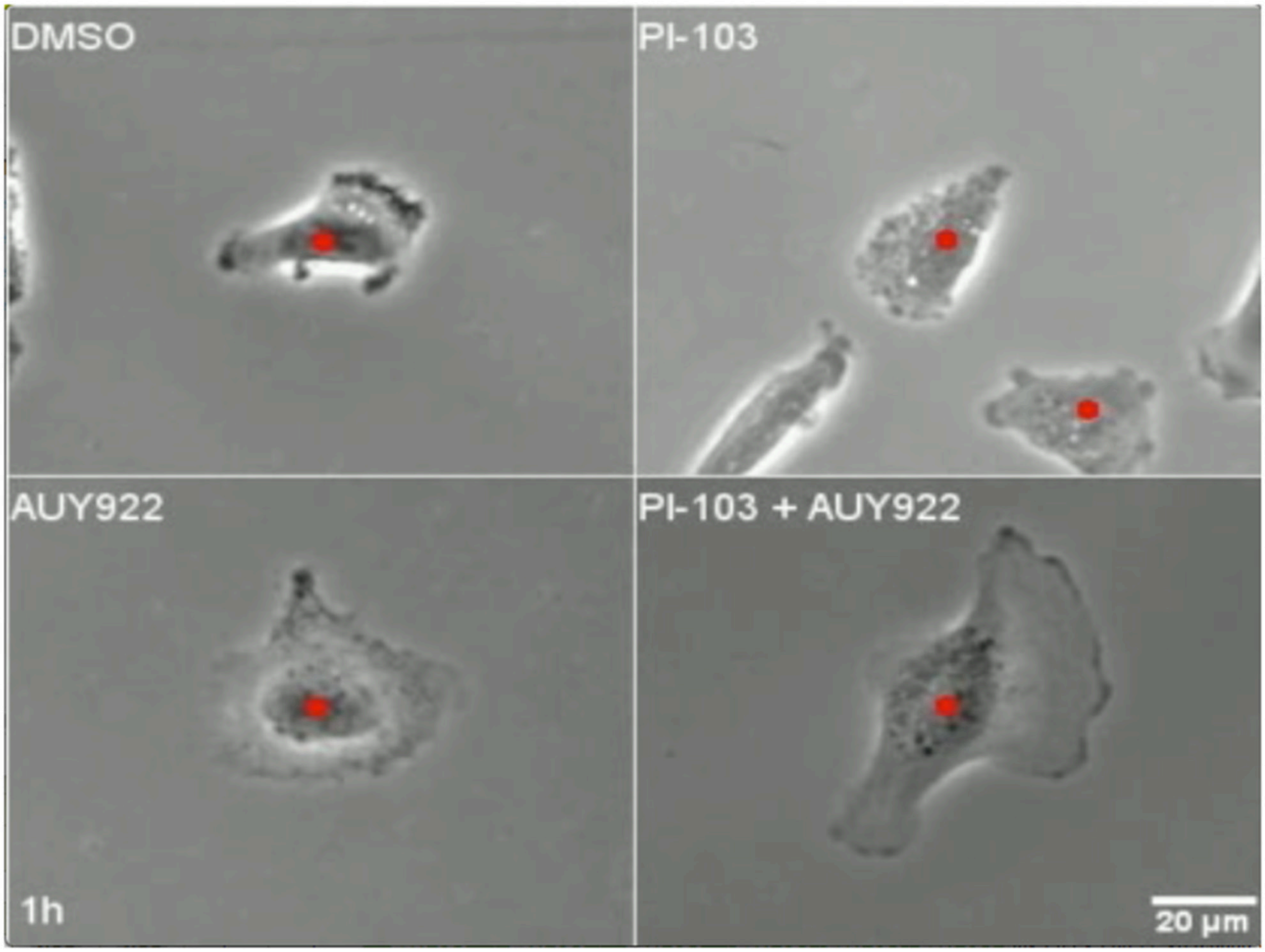

Supplementary Video 2: SNB19 cells were imaged every 10 min by time-lapse microscopy using a Nikon BioStation IM-Q (Nikon, Melville, NY). Prior to single-cell tracking experiments, about 10^4 cells were plated into a Petri dish (diameter 35 mm) containing 2 ml complete growth medium. In each experiment, time-lapse images were acquired over an 18-h period, using a ×10 phase contrast objective. Control DMSO-treated cells are shown in the top left movie. Drug-treated cells were pretreated 3 h before recording either with PI-103 (top right movie), AUY922 (bottom left movie) or a combination of both inhibitors (bottom right movie). The movies are played back at a rate of 15 frames per second (speed up 9000× real time).

See Supplementary Video 2
